# GPS Tracking of Free-Ranging Pigs to Evaluate Ring Strategies for the Control of Cysticercosis/Taeniasis in Peru

**DOI:** 10.1371/journal.pntd.0004591

**Published:** 2016-04-01

**Authors:** Ian W. Pray, Dallas J. Swanson, Viterbo Ayvar, Claudio Muro, Luz M. Moyano, Armando E. Gonzalez, Hector H. Garcia, Seth E. O’Neal, Cysticercosis Working Group in Peru

**Affiliations:** 1 School of Public Health, Oregon Health and Science University, Portland, Oregon, United States of America; 2 Center for Global Health Tumbes, Universidad Peruana Cayetano Heredia, Tumbes, Peru; 3 Inserm Neuroépidémiologie Tropicale (NET), Institut d'Epidémiologie et de Neurologie Tropicale (IENT), Faculté de Médecine de l'Université de Limoges, Limoges, France; 4 Office of Training and Research. Regional Hospital II-2, Tumbes, Peru; 5 School of Veterinary Medicine, Universidad Nacional Mayor de San Marcos, Lima, Peru; 6 Department of Microbiology, Universidad Peruana Cayetano Heredia, Lima, Peru; University of Queensland School of Veterinary Science, AUSTRALIA

## Abstract

**Background:**

*Taenia solium*, a parasitic cestode that affects humans and pigs, is the leading cause of preventable epilepsy in the developing world. *T*. *solium* eggs are released into the environment through the stool of humans infected with an adult intestinal tapeworm (a condition called taeniasis), and cause cysticercosis when ingested by pigs or other humans. A control strategy to intervene within high-risk foci in endemic communities has been proposed as an alternative to mass antihelminthic treatment. In this ring strategy, antihelminthic treatment is targeted to humans and pigs residing within a 100 meter radius of a pig heavily-infected with cysticercosis. Our aim was to describe the roaming ranges of pigs in this region, and to evaluate whether the 100 meter radius rings encompass areas where risk factors for *T*. *solium* transmission, such as open human defecation and dense pig activity, are concentrated.

**Methodology/Principal Findings:**

In this study, we used Global Positioning System (GPS) devices to track pig roaming ranges in two rural villages of northern Peru. We selected 41 pigs from two villages to participate in a 48-hour tracking period. Additionally, we surveyed all households to record the locations of open human defecation areas. We found that pigs spent a median of 82.8% (IQR: 73.5, 94.4) of their time roaming within 100 meters of their homes. The size of home ranges varied significantly by pig age, and 93% of the total time spent interacting with open human defecation areas occurred within 100 meters of pig residences.

**Conclusions/Significance:**

These results indicate that 100 meter radius rings around heavily-infected pigs adequately capture the average pig’s roaming area (i.e., home range) and represent an area where the great majority of exposure to human feces occurs.

## Author Summary

*Taenia solium*, commonly known as the pork tapeworm, is a parasite that affects humans and pigs. It has a disproportionate impact on low and middle income countries, and is most common in rural areas where free-ranging domestic pigs are common, and access to sanitation is limited. Pigs acquire cysticercosis, the larval stage of the disease, when they ingest *T*. *solium* eggs that have been released into the environment through the feces of an infected human host. In this study, we tracked free-ranging pigs with GPS devices, and found that most pigs remained close to their owners’ homes throughout the tracking period, and that the majority of pigs’ interactions with open human feces occurred near their owners’ homes. These findings suggest that 100 meter radius rings around heavily-infected pigs capture the most likely area of pig infection, and support focused control interventions in areas surrounding heavily-infected pigs.

## Introduction

*Taenia solium*, the pork tapeworm, is responsible for taeniasis and cysticercosis in humans. *T*. *solium* is highly endemic in many low and middle income countries, affecting 50 million people worldwide [1]. Neurocysticercosis (NCC), a condition that occurs when *T*. *solium* larval cysts infect the human central nervous system (CNS), is the leading cause of preventable epilepsy in the developing world, accounting for 30% of seizure disorders in these countries [2–5]. In Latin America alone, between 400,000 and 1.3 million people have epilepsy due to NCC [6].

Humans are the definitive host of the adult intestinal tapeworm, a condition known as taeniasis. *T*. *solium* eggs or gravid proglottid segments containing 50,000 or more infectious eggs are expelled periodically through the feces of humans with taeniasis [7]. In rural regions where open human defecation is common, these eggs or gravid proglottids contaminate the environment, and are consumed by free-ranging domestic pigs. Upon ingestion of *T*. *solium* eggs, pigs become infected with intermediate stage larval cysts in their tissues, (porcine cysticercosis). To complete the life-cycle, humans may then be infected with taeniasis by ingesting these cysts in undercooked pork. Human cysticercosis and NCC occur when humans accidentally ingest *T*. *solium* eggs from the feces of humans with taeniasis. The eggs then develop into cysts, which cause pathology in human tissue, including the brain and other parts of the CNS.

Previous attempts to eliminate cysticercosis and taeniasis from communities have focused on mass chemotherapeutic treatment of humans and pigs [8–10]. Mass treatment, however, is inefficient, as the prevalence of taeniasis is low even in endemic areas (1–2%) [11–13], meaning that the vast majority of treatments are applied to individuals without taeniasis. Risks associated with anti-parasitic treatment are also largely assumed by those individuals without disease [14]. Further, even mass treatment programs that have been met with initial success have not resulted in sustained reductions in transmission without repeated interventions [15]. This could be due to incomplete participation, or a failure to account for underlying environmental risk factors.

A control strategy to intervene within select high-risk foci has been proposed as an alternative to mass treatment [16]. In this ring strategy, humans are screened and/or treated for taeniasis if they live within 100 meters of a pig heavily-infected with cysticercosis, as identified by tongue examination [17,18]. The basis for this approach was a single small study in rural Peru that found the prevalence of taeniasis to be eight times greater among humans living within 100 meter radii of heavily infected pigs [19]. Potential advantages of this approach are higher levels of participation from residents living within high-risk foci, reduced costs, and sustainability as a permanent, locally-administered program. Despite this, 100 meter radius rings around infected pigs have never been validated as accurate representations of pig range in Latin America. GPS collars have recently been used to evaluate the ranges of free-roaming pigs in western Kenya [20], but we believe that our study remains an important and unique contribution, given the differences in geography and animal husbandry that may exist between the two regions. Moreover, few studies have evaluated the locations of open human defecation areas in relation to pig roaming areas [21], and none have done so with the precision allowed by the use of GPS technology. Evaluating the relative locations of pig roaming areas and open human defecation areas is an important step in determining where pigs may be exposed to the greatest risk of infection from *T*. *solium* eggs. The objectives of this study, therefore, were to use GPS tracking technology to describe the roaming ranges of rural pigs in this region, and to evaluate whether the 100 meter radius rings encompass areas where risk factors for *T*. *solium* transmission, such as open human defecation and pig activity, are concentrated.

## Methods

### Study Site and Participants

**Fig 1:**
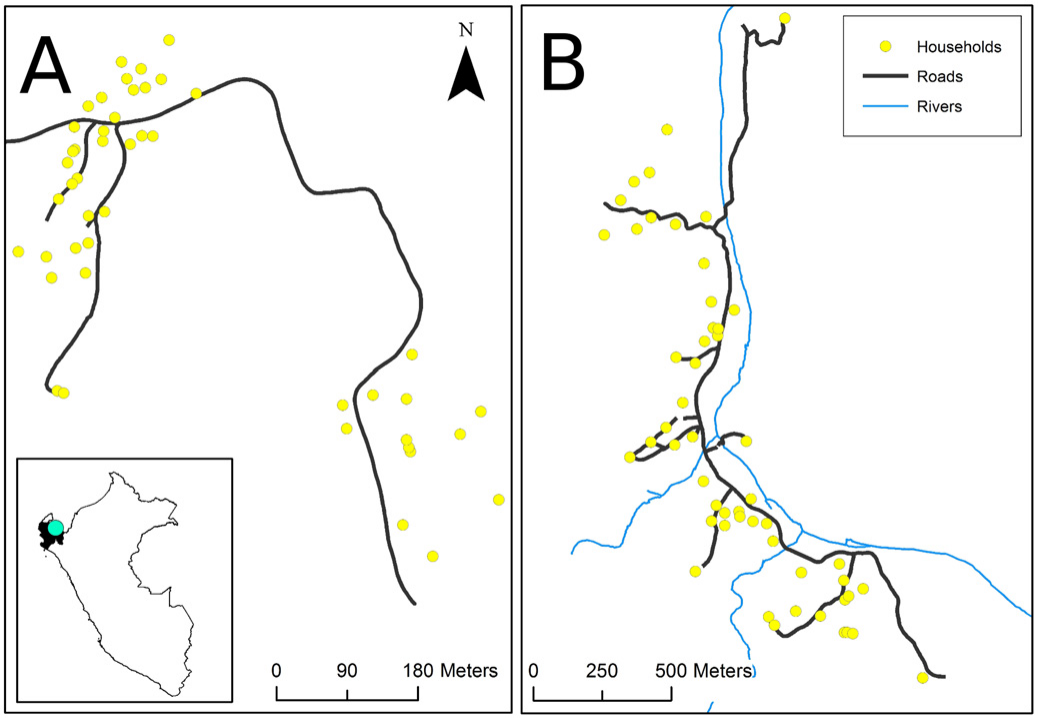
Map of two study villages located in the state of Piura, Peru. (A) Village of Minas de Jambur, population 140, 17 pigs tracked; (B) Village of Cachaco, population 132, 20 pigs tracked.

Two villages in the northern Peruvian region of Piura were selected for inclusion in this study (Fig 1). We selected the two villages, Minas de Jambur and Cachaco because they were non-intervention villages participating in a concurrent ring treatment trial. Further, their small populations allowed us to attempt to select at least one pig for GPS tracking from every pig-owning household, thereby achieving adequate spatial representation of pigs throughout the villages. Pigs were eligible for inclusion if they were between 2 and 24 months of age, and were not pregnant, nursing, or sick at the time of the visit. Among the eligible pigs, we enrolled one male and one female from each household when possible. When more than one eligible male and female pig were available at each household, we enrolled the first pig captured by the field team.

### GPS Tracking of Pigs

**Fig 2:**
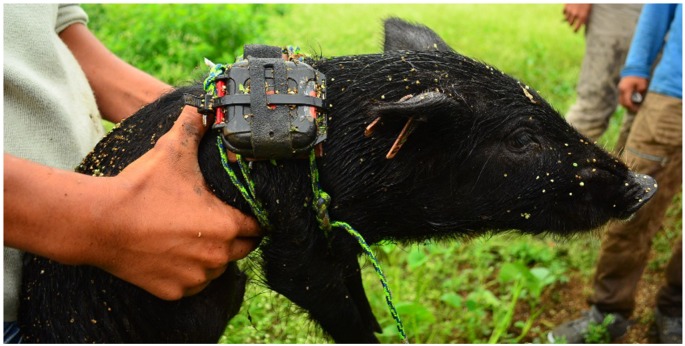
GPS device secured properly to pig upon capture.

Once eligible pigs were captured, one “iGotU” portable GPS receiver (MobileAction Technology, New Taipei City, Taiwan) was turned on, placed in a waterproof case (HPRC 1100, Plaber, Vicenza, Italy), and secured to a leather harness on the nape of the pig (Fig 2). We programmed the GPS receivers to log the geographic coordinates of the pig’s location every 30 seconds. After 48 hours, we returned to enrolled households, captured the pigs, removed the GPS data loggers, and downloaded the data points.

### Household Defecation Survey

In addition to tracking pigs, we visited every household in the village to assess household defecation practices. We asked available adult residents to indicate where household members preferred to defecate. If outdoor areas were indicated, we used handheld GPS receivers (GeoExplorer II; Trimble, Sunnyvale, CA) with post-processed differential correction for sub-meter accuracy to log perimeters around the indicated areas. If there was a latrine on the residence, we logged a single point in front of the latrine. For latrines and outdoor defecation areas, household respondents were asked to rate their frequency of use–“Never,” “Sometimes,” or “Always.” Finally, survey teams inspected roads, riverbeds, and communal areas such as schools, churches, and athletic fields for evidence of open human defecation. If human feces were identified, GPS points were logged at their locations.

### Mapping and Statistical Analysis

All data were analyzed using Stata version 13.1 (StataCorp; College Station, TX), R version 3.1 (The R Foundation for Statistical Computing; www.r-project.org), and ArcGIS version 10.2 (ESRI; Redlands, CA). Because obstruction or disruption of the satellite signal occurred intermittently during pig roaming, thereby affecting GPS precision, we utilized the following protocol for removing outlying points. We excluded points that were in the top 1% of speeds measured throughout the tracking period for each pig. The excluded percentile typically consisted of speeds that could not plausibly be attributed to true pig movement. Additionally, we excluded all points taken after a substantial time lag (upper 1% of time lags for each pig). In order to reduce the impact of the chase and capture of the pig on their movement and behavior, we excluded the first hour and last 15 minutes of logged points. A mean of 294 points, or 6.7% of data points logged for each pig, were excluded due to suspected error.

We analyzed pig roaming ranges using the “LoCoH” (Localized Convex Hulls) Homerange Analysis Algorithm for R [22,23]. We used the fixed *k* LoCoH method, which works by creating convex polygon hulls from the *(k-*1) nearest neighbors around each GPS point. A detailed protocol for the fixed *k* LoCoH method can be found elsewhere [24]. In brief, we set the value of *k* to 30 for all pig ranges, as this produced the most spatially precise ranges while maintaining smooth and contiguous areas. Convex polygon hulls from the 30 nearest neighbors around each point were sorted, stacked from smallest to largest, and merged to form desired isopleth levels. The output of the fixed *k* LoCoH method was a plot of each pig’s range identifying three areas based on the specified isopleth cut-off values: the “core utilization area” represented the densest 50% of pig range; the “home range” represented the densest 90% of pig range; and the “maximum range” was the area that contained 100% of the convex hulls. Homerange shapefiles produced in R were then exported to ArcGIS 10.2 for spatial analysis of home ranges as they corresponded to buffers created around each household, and open defecation areas.

GPS coordinates for households, latrines, and open defecation areas were imported to ArcGIS for use in the analysis. For individual defecation points found in the community, we created five meter buffers to represent areas of potential contamination. We created 50, 100, 150, and 200 meter buffers around each pig-raising household to assess pig ranges with respect to the rings. We calculated the proportions of pig tracking points that were located inside and outside of the rings for each pig, along with the proportions of GPS points located inside mapped open defecation areas for each pig.

### Ethics

This study was reviewed and approved by the Institutional Review Boards at the Universidad Peruana Cayetano Heredia (UPCH) and at Oregon Health & Science University (OHSU). All adult participants provided written informed consent. The study was also reviewed by the Institutional Ethics Committee for the Use of Animals at UPCH as well as the Institutional Animal Use and Care Committee at OHSU. Treatment of animals adhered to the Council for International Organizations of Medical Sciences (CIOMS) International Guiding Principles for Biomedical Research Involving Animals.

## Results

### Swine Population and Range

There were 77 inhabited households and an estimated total of 336 pigs raised among the two villages surveyed. 44% of the inhabited households reported raising pigs at the time of the survey. The median number of pigs raised per pig-raising household was six.

41 pigs from the two villages were tracked over the course of the study period. GPS coordinates from four of the devices were excluded because the devices came loose from the pigs during the tracking period or were found to have produced coordinates with substantial measurement error. This led to a final analytic sample of 37 pigs from the two villages. Among the pigs analyzed, the median age, as estimated by the owner, was 6 months (range 2–24 months), with a median estimated weight of 12 kilograms (range 6–40 kilograms). 19 (51%) of the pigs tracked were female. Of the 18 male pigs tracked, 8 (44%) were castrated. Characteristics of pigs tracked are summarized by village in Table 1.

**Table 1:** Characteristics of pigs tracked in Minas de Jambur and Cachaco, Peru.

Pig tracking characteristics, n = 37		Village	
		Minas de Jambur (n = 17)	Cachaco (n = 20)
Sex [no. (%)]	Male (complete)	2 (11)	8 (40)
	Male (castrated)	8 (47)	0 (0)
	Female	7 (41)	12 (60)
Estimated age, months [no. (%)]	2–4	5 (29)	5 (25)
	5–6	8 (47)	6 (30)
	7–9	0 (0)	5 (25)
	12–24	4 (24)	4 (20)
Estimated weight, kg [median (range)]		12 (10–40)	15 (6–40)

**Fig 3:**
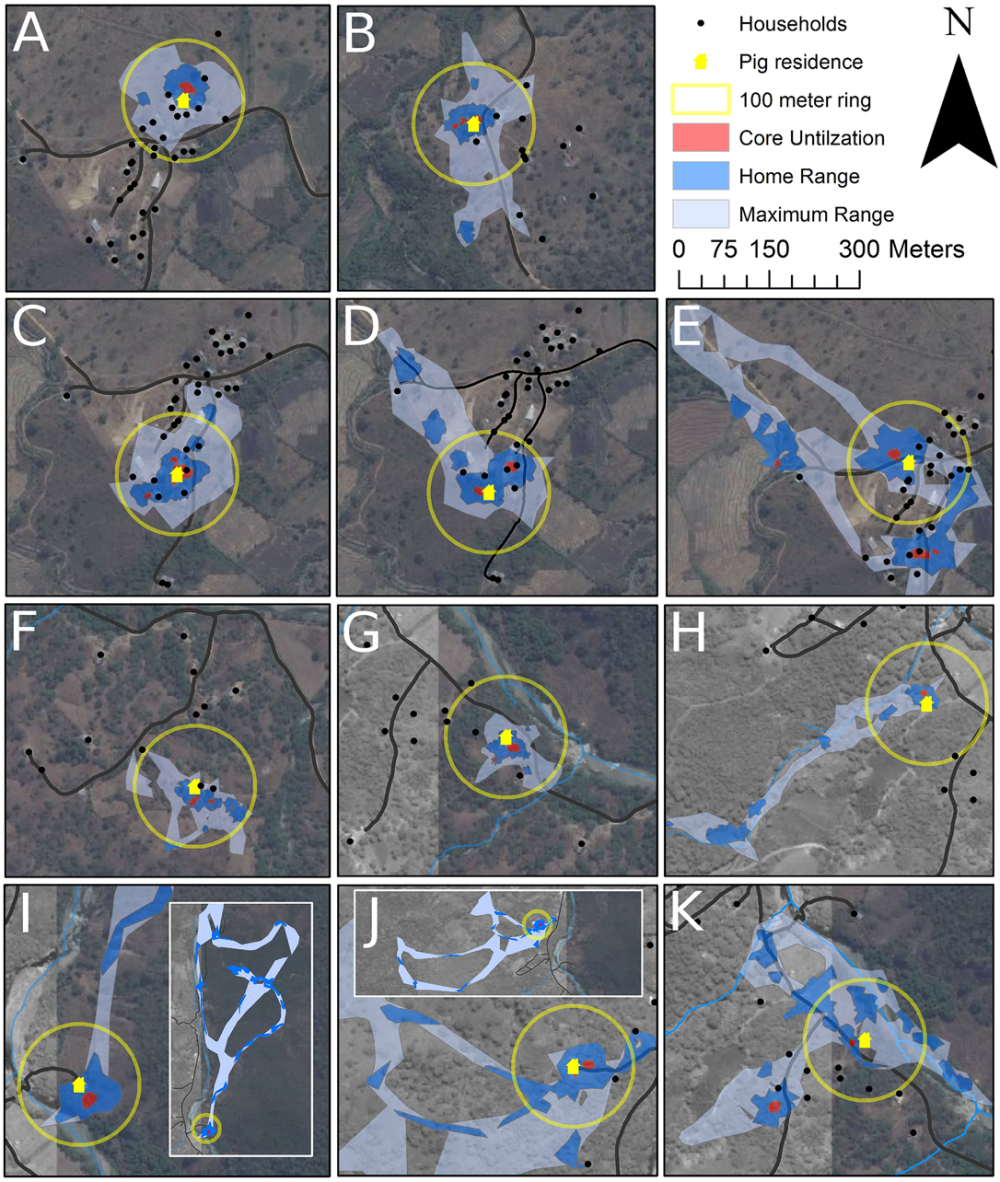
Roaming ranges and 100 meter radius rings around pig residences for 11/37 pigs tracked. Minas de Jambur: (A) 4 month old castrated male pig with range heavily concentrated within 100m radius ring; (B) 12 month old female pig with a few short trips out of the 100m radius ring into vegetation and along road; (C, D) 6 and 5 month old female pigs with range hotspots next to both own and neighboring residences within 100m; (E) 12 month old male pig with dispersed range including hotspot behind own house, and among distant houses. Cachaco: (F) 6 month old female pig with range concentrated behind both own and neighboring residences within 100m; (G) 3 month old male pig with range heavily concentrated within 100m radius ring; (H) 3 month old male pig with hotspot next to residence, and trips outside of 100m radius ring following riverbed; (I,J) 8 month old male and female pigs with hotspots behind residence, but including multiple extended trips into vegetation outside of 100m radius ring; (K) 5 month old male pig with one hotspot near residence, and a second large hotspot at a neighboring residence outside of 100m radius ring. Service Layer Credits: Source: ESRI, DigitalGlobe, GeoEye, Earthstar Geographics, CNES/Airbus DS, USDA, USGS, AEX, Getmapping, Aerogrid, IGN, IGP, swisstopo, and the GIS User Community.

The area of home range and core utilization were determined for each pig. Summary measures are shown in Table 2. Home range differed significantly by pig age, while core utilization differed by village. Home range increased with age up to 9 months old, then decreased in pigs 12 months or older. 7–9 month-old pigs (n = 5) had the largest home ranges of any age category (median: 35.5 km^2^; [range: 21.4, 55.9]), and were all from the village of Cachaco. Pigs from the village of Cachaco also had a larger median home range than pigs from Minas de Jambur, although the difference was not significant. In contrast to our findings with home ranges, median core utilization areas were larger in Minas de Jambur (0.52 km^2^; range 0.21, 1.5) than in the village of Cachaco (0.35 km^2^; range 0.13, 0.71), but did not vary significantly by any other characteristic. Maximum range, the areas that comprises the full 100% of a pig’s range, was highly correlated with home range for each pig (r = 0.88). Fig 3 shows the core utilization areas, home ranges, and maximum ranges for 11 representative pigs of the 37 pigs tracked. Range maps for all other pigs, along with open human defecation areas, can be found in the appendices (S1 and S2 Map Appendices).

**Table 2:** Relationship between pig characteristics, pig roaming ranges, and interactions with open human defecation areas.

Characteristics		Number of pigs (n = 37)	Area of Home Range (km^2^)*	Area of Core Utilization (km^2^)*	Minutes per day interacting with open defecation areas*
Village	Cachaco	20	13.9 (5.0, 22.3)	0.35 (0.27, 0.47)^	5.1 (0.9, 10.7)
	Minas de Jambur	17	6.3 (5.0, 10.9)	0.52 (0.46, 0.81)^	29.6 (7.5, 65.0)
Sex	Male (complete)	10	12.6 (4.8, 21.9)	0.38 (0.22, 0.54)	6.7 (1.0, 9.1)
	Male (castrated)	8	6.2 (5.4, 7.4)	0.50 (0.47, 0.71)	20.3 (0.9, 126.6)
	Female	19	11.2 (5.2, 21.4)	0.39 (0.30, 0.70)	9.8 (0.9, 42.1)
Age (months)	2–4	10	4.8 (2.7, 6.4)^	0.29 (0.21, 0.67)	6.4 (1.0, 29.6)
	5–6	14	9.3 (6.1, 16.9)^	0.49 (0.39, 0.73)	29.3 (7.4, 47.9)
	7–9	5	35.5 (22.7, 40.3)^	0.49 (0.33, 0.51)	8.4 (2.1, 9.1)
	12–24	8	10.5 (6.8, 17.3)^	0.47 (0.34, 0.59)	1.0 (0.2, 36.9)

*Median and interquartile range (IQR) reported

^Kruskal-Wallis non-parametric p-value < 0.05

### Pig Range and Rings

**Fig 4:**
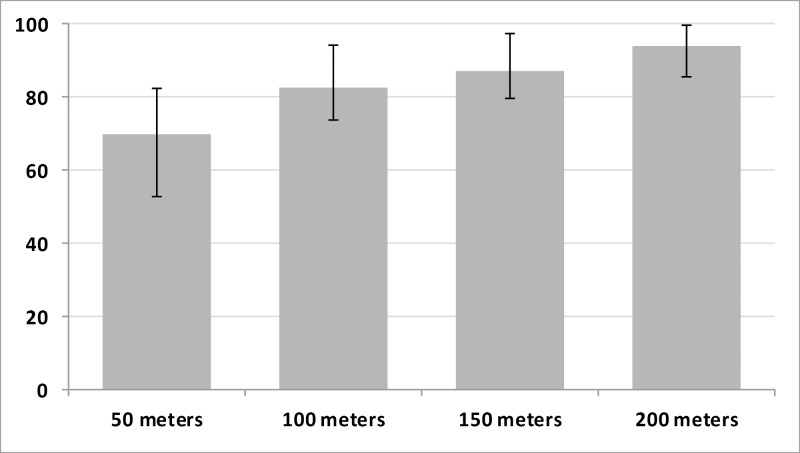
Percent of tracking period within 50, 100, 150, and 200 meters of pig residence, (median [IQR]).

Pigs spent a median of 82.8% (IQR: 73.6, 94.4) of the tracking period within 100 meters of their owner’s residence (Fig 4). However, there was considerable heterogeneity among pigs as the percent of time spent inside the 100 meter radius ring ranged from a minimum of 26% to a maximum of 99% for different pigs. In comparison, the 50 meter radius ring contained a median of 69.8% (IQR: 52.3, 82.4) of the tracking period, while the 150 and 200 meter radius rings contained medians of 87.0% (IQR: 79.5, 97.2) and 93.8% (IQR: 85.5, 99.6) of pig tracking periods, respectively.

### Human Population and Defecation Survey

We successfully interviewed adult residents about their defecation practices in 71 (92%) of the 77 households among the two villages. 21 (27%) households did not have latrines on their properties. Outdoor defecation areas were logged for 19 out of 21 households without latrines. Among the 56 households with latrines, 14 (25%) reported using open defecation areas at least some of the time. In total, 36 (47%) of the household reported using open defecation areas at least some of the time. Our field team also identified 34 open defecation areas within the communities that could not be identified as belonging to a particular household. Evidence of open human defecation was found along dry river beds and along paths through the communities. In one of the villages, a heavily used open defecation area was found along a path leading to family farm plots that had been repeatedly used by many as a communal defecation area. In all, 65 open defecation areas were logged between the two villages, 34 unidentified fecal remains, and 31 areas identified by household members. The two villages had approximately equal total combined areas of open defecation.

### Pig Interactions with Defecation Sites

The amount of time pigs spent interacting with open defecation areas did not differ significantly by any measured factor, however pigs in the village of Minas de Jambur spent a median of 29.6 (IQR: 7.5, 65.0) minutes per day interacting with open defecation areas, compared to just 5.1 (IQR: 0.9, 10.7) minutes per day for pigs from the village of Cachaco (Mann-Whitney p-value = 0.06) (Table 2). While age-specific differences in time spent interacting with open defecation areas were not significant, 5–6 month old pigs spent a median of 29.3 (IQR: 7.4, 47.9) minutes per day, while no other age category had a median greater than 10 minutes per day spent interacting with open defecation areas. Similar to the variability found in the area of pig ranges, there was considerable heterogeneity among pigs with respect to the amount of time spent interacting with open defecation area, which ranged from zero (4 pigs) to nearly four hours per day.

**Fig 5:**
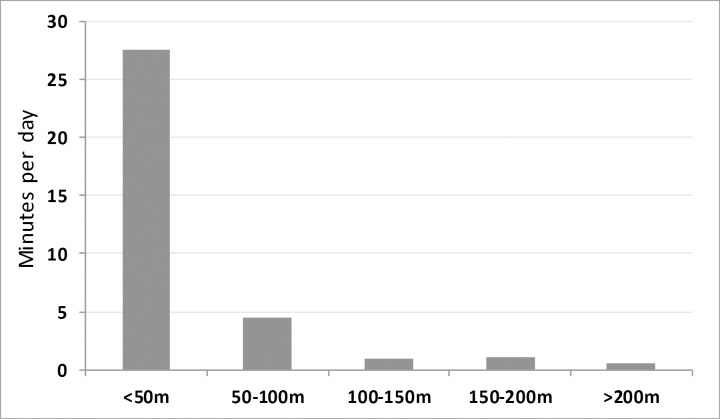
Minutes per day spent interacting with open defecation areas at increasing distances from pig residence, (mean).

The majority of pig interactions with open defecation areas occurred in close proximity to the pigs’ residences, with pigs spending a mean of 27.6 minutes per day interacting with open defecation areas within 50 meters of their residence. Pigs spent a combined mean of only 7.0 minutes per day interacting with open defecation areas located more than 50 meters from their residence (Fig 5). Overall, 93% of the total time pigs in this study spent interacting with open defecation areas occurred within 100 meters of their residence.

Pigs whose owners reported practicing open defecation spent a median of 42.1 (IQR: 8.4, 158.5) minutes per day interacting with open defecation areas, compared to just 2.0 (IQR: 0.3, 11.6) minutes per day among pigs whose owners reported using latrines only. Among pigs whose owners practiced open defecation, a median of 8.3 (IQR: 6.1, 47.7) minutes per day were spent interacting with the pigs’ owners’ open defecation areas, while a median of just 0.8 (IQR: 0, 12.7) minutes per day were spent interacting with neighboring open defecation areas. (Table 3).

**Table 3:** Minutes per day pigs spent interacting with home and neighboring open defecation areas, by household defecation practice.

	Household Defecation Practice		
	Open Field Defecation (n = 15)^§^	Latrine (n = 22)^§^	p-value*
All open field defecation areas	42.1 (8.4, 158.5)	2.0 (0.3, 11.6)	< 0.001
Open defecation areas within 100 meters of pig’s residence	42.1 (8.4, 107.1)	0 (0, 4.8)	< 0.001
Open defecation sites of pig’s residence	8.3 (6.1, 47.7)	NA	NA
Open defecation site of *neighboring* households within 100m of pig’s residence	0.8 (0, 12.7)	0 (0, 4.8)	0.24

*Kruskal-Wallis non-parametric p-value reported

§ median and IQR reported

## Discussion

The results of this study provide important insight into the size and location of roaming areas for free-ranging pigs in northern Peru, a region endemic for *T*. *solium* cysticercosis. We found that tracked pigs spent a median of 82.8% (IQR: 73.6, 94.4) of their time roaming within 100 meters of their homes. These findings indicate that 100 meter radius rings around heavily-infected pigs used in ring interventions cover the majority, but not entirety, of typical roaming ranges for pigs in this region. We also found that pigs spent a median of 29.6 (IQR: 7.5, 65.0) and 5.1 (IQR: 0.9, 10.7) minutes per day interacting with open human defecation areas in the two villages, with 93% of this time occurring within 100 meters of the pigs’ residence. While this study did not evaluate pig infection, and the possibility remains that pigs could be infected by *T*. *solium* eggs during roaming outside of the 100 meter radius rings, our findings suggest that the most likely area of infection for pigs in this region is within 100 meters of their residences, as risk factors for *T*. *solium* transmission were found to be concentrated in these areas. Our findings, therefore, support interventions that target their efforts in the areas immediately surrounding heavily-infected pigs, where *T*. *solium* transmission is most likely to occur.

The most important factor that predicted for increased interaction with open defecation areas was the defecation practice reported by the pig owner. Pigs that belonged to owners who practiced open defecation spent a median of 42.1 (IQR: 8.4, 158.5) minutes per day interacting with open defecation areas, while pigs belonging to owners that reported using latrines spent a median of 2.0 (IQR: 0.3, 11.6) minutes per day interacting with open defecation areas. There is strong evidence that open field defecation is a risk factor for porcine cysticercosis [25,26]. Our study suggests that open human defecation that takes place in the immediate surroundings of homes that raise free-ranging pigs may be significant sources of exposure to *T*. *solium* eggs and important routes of transmission to be considered in future control efforts.

Another important finding in this study was the influence of pig age on roaming behavior. Pigs 2 to 4 months old had significantly smaller home ranges than 7 to 9 month-old pigs. As a result of their limited ranges, we found that 2 to 4 month-old pigs spent more time interacting with open defecation areas than any other age category, as open defecation areas were typically located in close proximity to houses. This suggests that young pigs are more likely to be exposed to human feces, and may be at a higher risk of infection from *T*. *solium* eggs than older pigs. The idea of increased infection risk among younger pigs is consistent with one study done in a rural Peruvian village that found 4 out of 9 pigs born to seronegative sows in an endemic area had seroconverted by 5 months of age [27]. A study in Mozambique, however, opposed this finding by observing a lower incidence of serum antigens among younger pig [28]. A possible explanation for this lower incidence among young pigs, despite their increased exposure to *T*. *solium* eggs, could be the passive transfer of maternal antibodies, which may protect pigs at young ages [29,30], and the fact that cysts take time to establish, grow, and reach levels that would be detectable by antigen tests. It is also possible that, while younger pigs spend more time in open defecation areas, older dominant pigs outcompete younger pigs for access to open feces [21], therefore putting older pigs at increased risk for infection despite spending less time in open defecation zones.

The differing sizes of home ranges observed between the two villages, with pigs from Minas de Jambur exhibiting much smaller home ranges than pigs from Cachaco, were likely attributable to the unequal age distribution of pigs selected for tracking from the two villages. All five of the 7–9 month old pigs in this study were from the village of Cachaco, and, as noted above, these pigs had larger home ranges than any other age group. It is possible that other geographical characteristics of the villages, such as the dispersed nature of homesteads in the village of Cachaco compared to denser housing in Minas de Jambur, also contributed to the observed differences in home ranges.

A few important qualitative observations were made about the size and distribution of pig ranges in this study. First, 15 of the 16 pig pairs that were tracked from the same household had nearly identical roaming ranges. This correlation among pigs from the same household confirms our understanding of pigs as largely herd animals that roam and forage together [21], and may suggest that pigs of the same household share similar exposure profiles in relation to *T*. *solium* eggs and human feces. Additionally, we observed that when the pigs did leave their homestead and venture through the village, they traveled primarily along road, paths, and streams. This suggests that efforts to identify and mitigate potential environmental exposures to *T*. *solium* eggs should be focused around the households, and along streams and thoroughfares frequented by pigs.

Result similar to those presented here were found by Thomas et al. [20], who used a home range analysis to evaluate roaming areas for 10 free ranging pigs in western Kenya. Thomas et al. found that the mean size of home ranges were very similar (15.1km^2^ vs. 14.5km^2^), and core utilization areas were larger (0.95km^2^ vs. 0.52km^2^) than those found in our study. They also found that pigs spent the majority (53%, on average) of their time roaming within the small confines of the family homestead. While Thomas et al. interpreted their results as evidence that pigs may be exposed to infectious agents such as *T*. *solium* eggs across wide and dispersed areas, we believe that both the findings of Thomas et al. and our own study support a hypothesis that exposure to *T*. *solium* eggs in this setting generally occurs in focalized areas around the pig residence, and that these areas can be roughly approximated with 100 meter radius rings around the residence. Of course, to test this hypothesis, further research must be undertaken that demonstrates the presence of *T*. *solium* eggs in the environment surrounding pig residences. Differing conclusions notwithstanding, the similarities in home range found between free-ranging pigs of Kenya and Peru support the generalizability of the results of this study to other regions where free-range pig raising is common and cysticercosis is prevalent.

There are a few important limitations in our study. First, the length of the tracking period (48 hours) was limited by the short battery life of the GPS devices used. This narrow window of tracking time is vulnerable to chance events, and to intervention effects, as pig behavior is likely to be affected by the stress of capture and restraint during the harnessing process. A longer tracking period would help to reduce the impact of those events. Similarly, the present study tracked pigs during the wet season only, during which pigs are more likely to roam further from their home areas to forage for available food sources [21]. Tracking should be repeated at least twice throughout a calendar year to account for seasonal variability. In addition, further tracking studies must include a larger sample size of pigs to aid in statistical power and reduce the impact of chance events. Lastly, in order to draw meaningful conclusions about possible risk factors for cysticercosis infection, it is important that future studies include serology of pigs to test for cysticercosis. It is important that we make the fundamental comparisons between exposed pigs, exposed but not infected pigs, and infected pigs when interpreting pig ranges in order to better understand which environmental exposures may be true risk factors for disease.

## Supporting Information

S1 Map Appendix Range maps for all 20 pigs tracked from the village of Cachaco, Peru. (PDF)

S2 Map Appendix Range maps for all 17 pigs tracked from the village of Minas de Jambur, Peru. (PDF)

S1 Dataset Characteristics of all pigs tracked. (XLSX)

S2 Dataset GPS coordinates for all pigs tracked during two-day tracking period in the village of Cachaco, Peru. (ZIP)

S3 Dataset GPS coordinates for all pigs tracked during two-day tracking period in the village of Minas de Jambur, Peru. (ZIP)

S4 Dataset Characteristics of households in Cachaco and Minas de Jambur, Peru. (XLSX)

S5 Dataset Shapefiles for town features in Cachaco, Peru (includes household coordinates, roads, streams, open defecation areas, corrals, and latrines). (ZIP)

S6 Dataset Shapefiles for town features in Minas de Jambur, Peru (includes household coordinates, roads, streams, open defecation areas, corrals, and latrines). (ZIP)
