# Supplementary material for: GPS Tracking of Free-Ranging Pigs to Evaluate Ring Strategies for the Control of Cysticercosis/Taeniasis in Peru
Source: PLoS Negl Trop Dis. 2016 Apr 1;10(4):e0004591. doi: 10.1371/journal.pntd.0004591 (PMC4818035; doi:10.1371/journal.pntd.0004591)
Supplement: S1 Map Appendix — (PDF) [file pntd.0004591.s001.pdf]

S1 – Map Appendix
Village of Cachaco, Piura, Peru

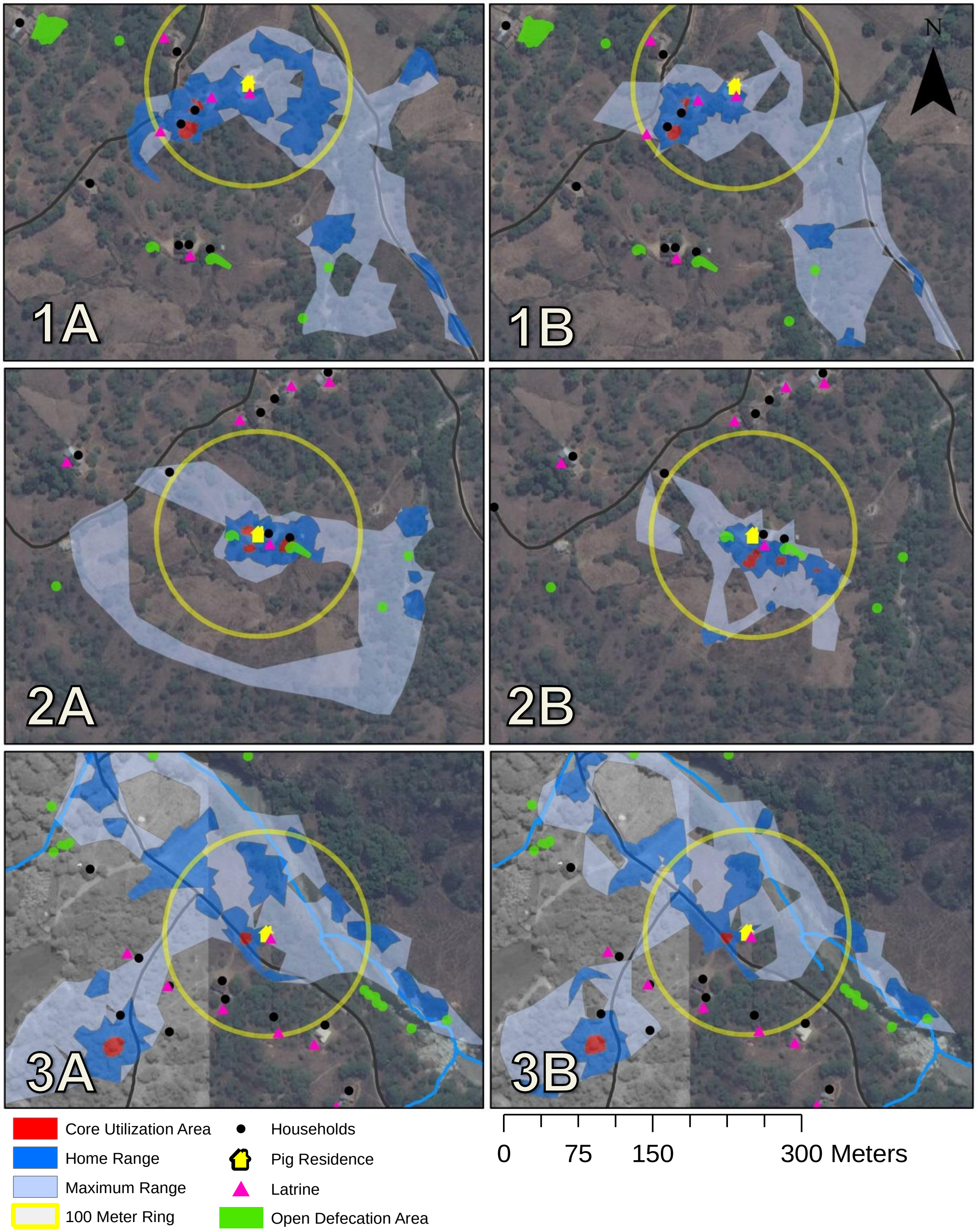

Pigs Tracked

Household 1: (1A) 24 month-old female; (1B) 2 month-old male
Household 2: (2A) 18 month-old female; (2B) 6 month-old female
Household 3: (3A) 5 month-old male; (3B) 5 month-old female

S1 – Map Appendix
Village of Cachaco, Piura, Peru

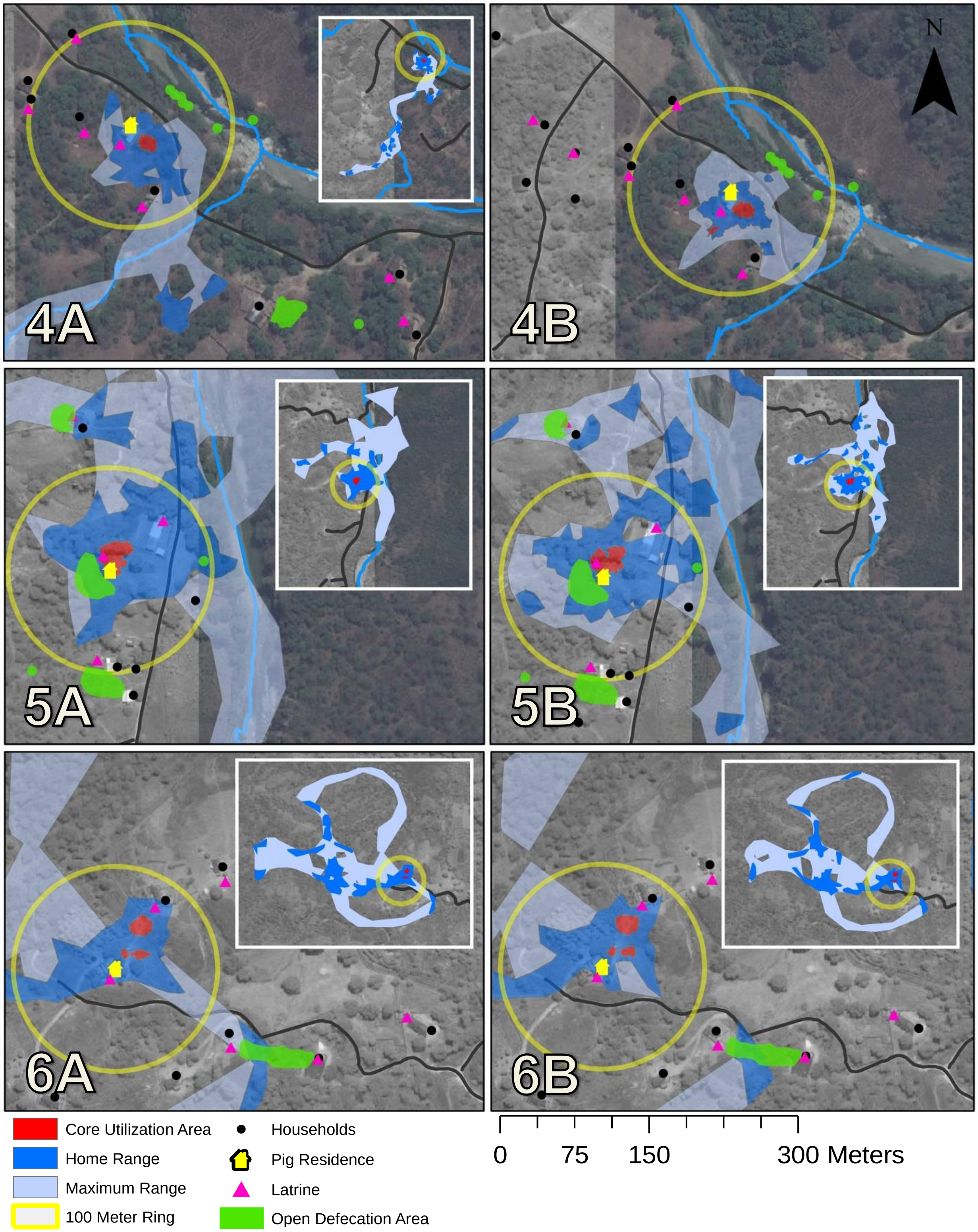

<u>Pigs Tracked</u>

Household 4: (4A) 18 month-old female; (4B) 3 month-old male
Household 5: (5A) 6 month-old female; (2B) 18 month-old female
Household 6: (6A) 8 month-old male; (3B) 8 month-old male

S1 – Map Appendix
Village of Cachaco, Piura, Peru

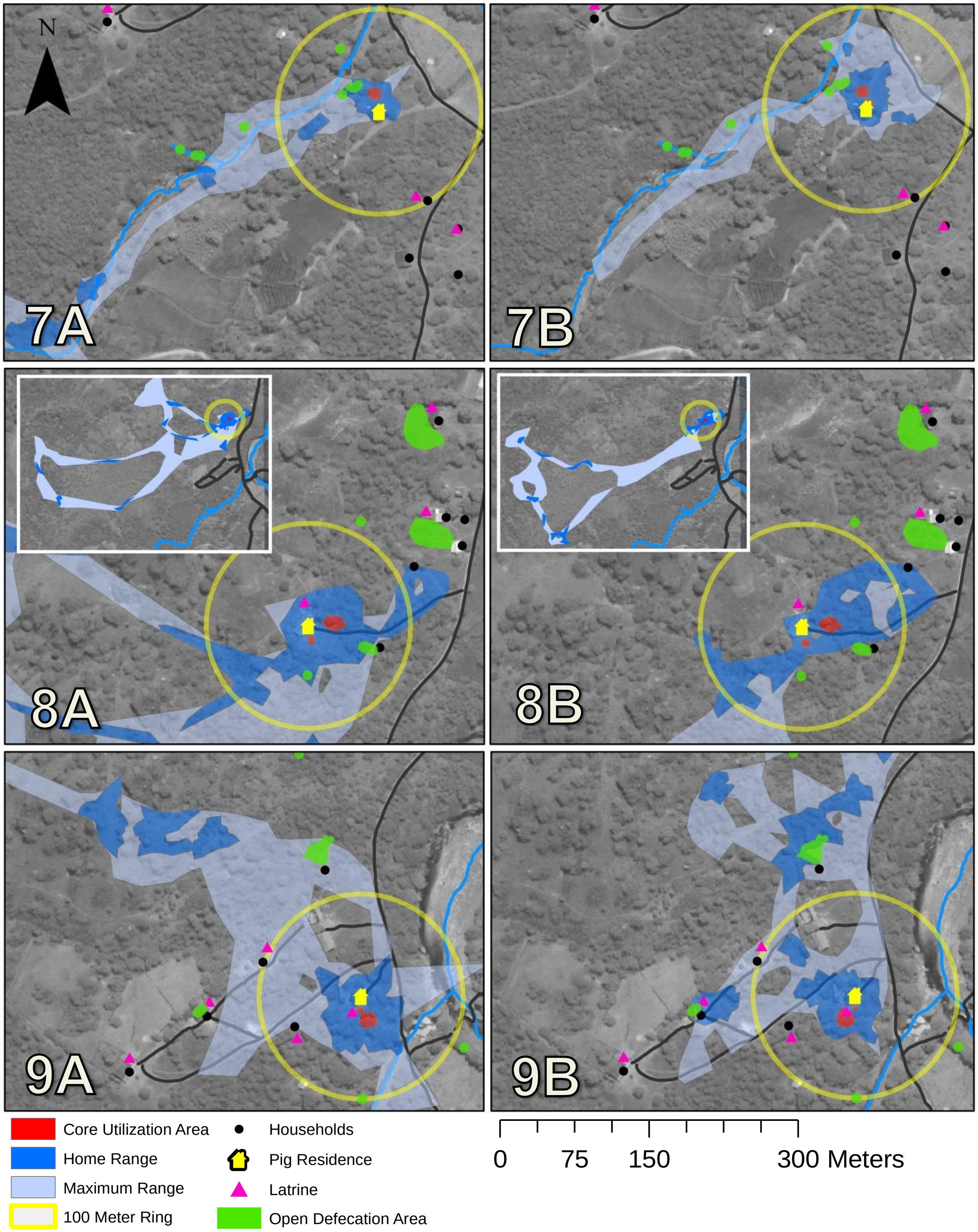

<u>Pigs Tracked</u>
Household 7: (7A) 3 month-old male; (7B) 3 month-old male
Household 8: (8A) 8 month-old female; (8B) 8 month-old female
Household 9: (9A) 4 month-old male; (9B) 18 month-old female

S1 – Map Appendix
Village of Cachaco, Piura, Peru

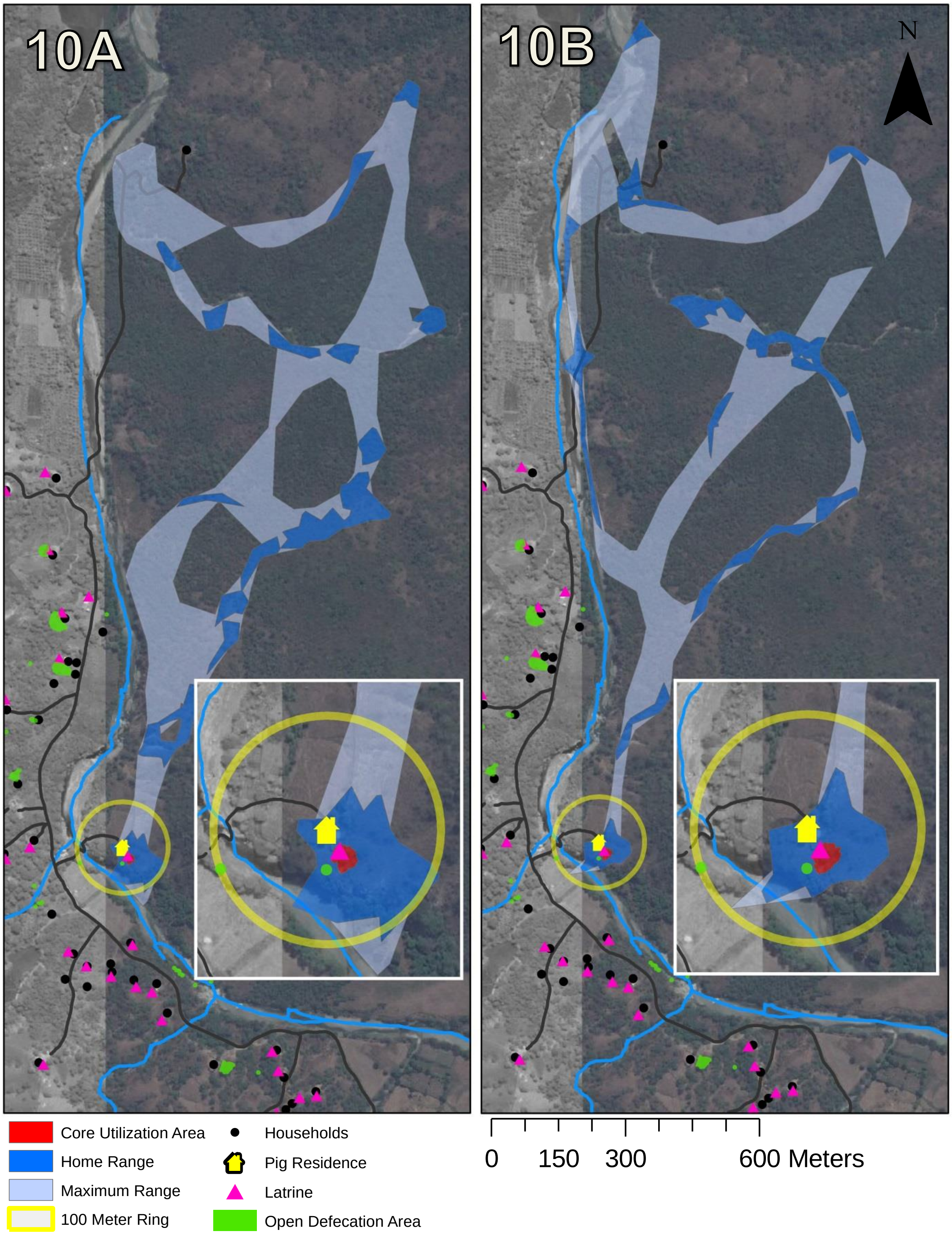

Pigs Tracked
Household 10: (10A) 6 month-old female; (10B) 8 month-old male
