# Supplementary material for: GPS Tracking of Free-Ranging Pigs to Evaluate Ring Strategies for the Control of Cysticercosis/Taeniasis in Peru
Source: PLoS Negl Trop Dis. 2016 Apr 1;10(4):e0004591. doi: 10.1371/journal.pntd.0004591 (PMC4818035; doi:10.1371/journal.pntd.0004591)
Supplement: S2 Map Appendix — (PDF) [file pntd.0004591.s002.pdf]

S2 – Map Appendix
Village of Minas de Jambur, Piura, Peru

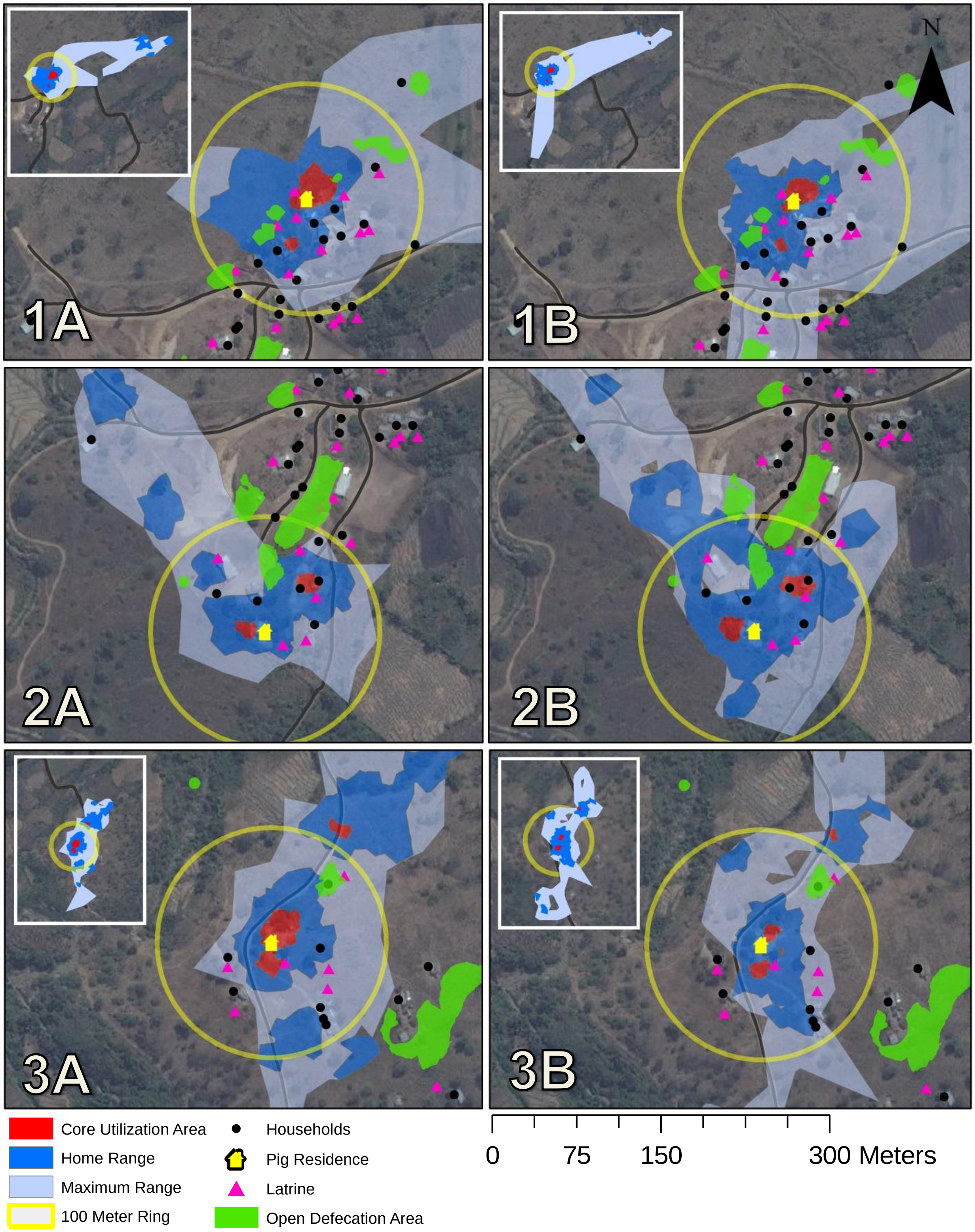

Pigs Tracked
Household 1: (1A) 6 month-old male; (1B) 6 month-old female
Household 2: (2A) 5 month-old female; (2B) 5 month-old male, castrated
Household 3: (3A) 4 month-old female; (3B) 12 month-old male, castrated

S2 – Map Appendix
Village of Minas de Jambur, Piura, Peru

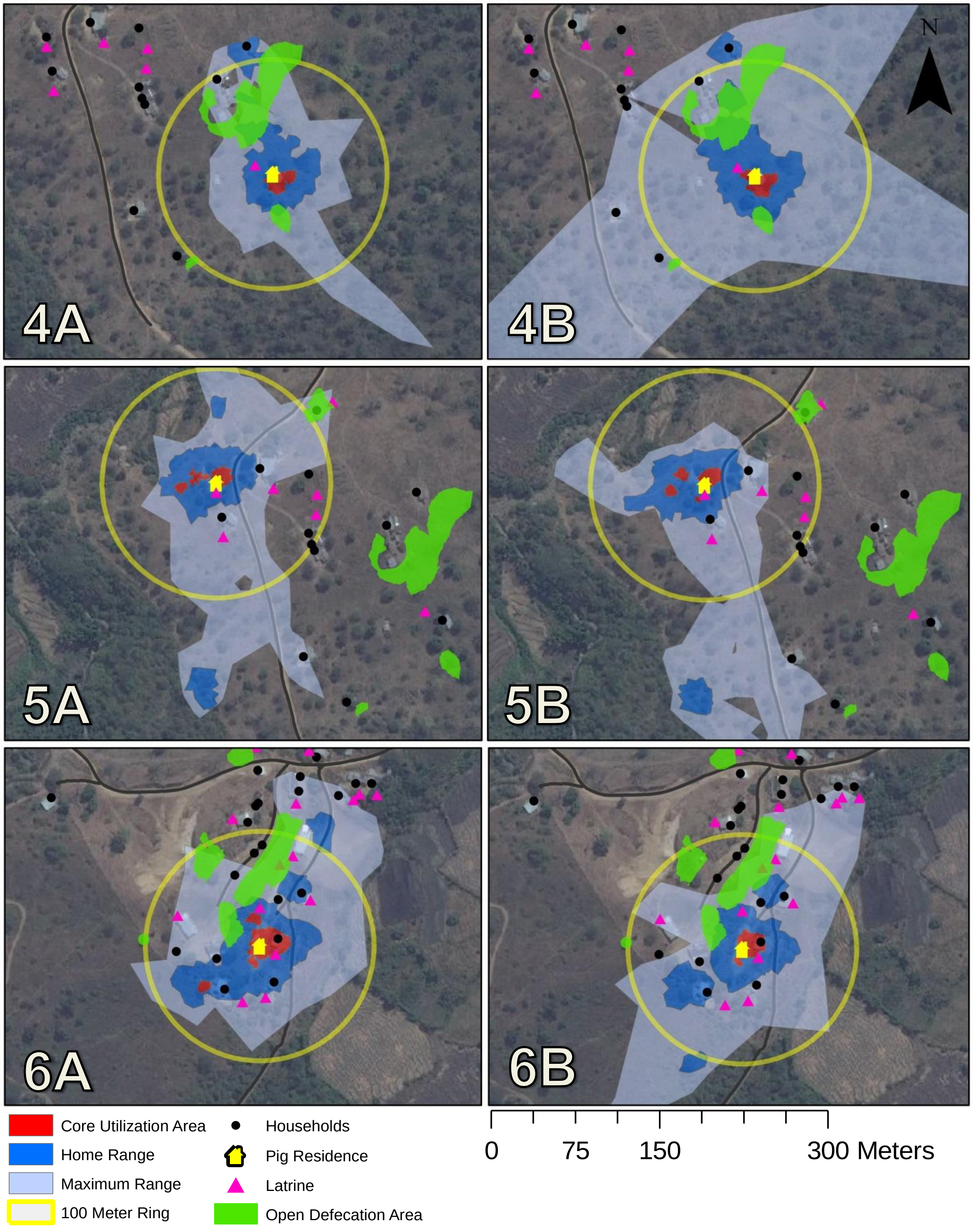

Pigs Tracked
Household 4: (4A) 6 month-old female; (1B) 6 month-old male, castrated
Household 5: (5A) 12 month-old female; (5B) 12 month-old male, castrated
Household 6: (6A) 6 month-old female; (6B) 6 month-old male, castrated

S2 – Map Appendix
Village of Minas de Jambur, Piura, Peru

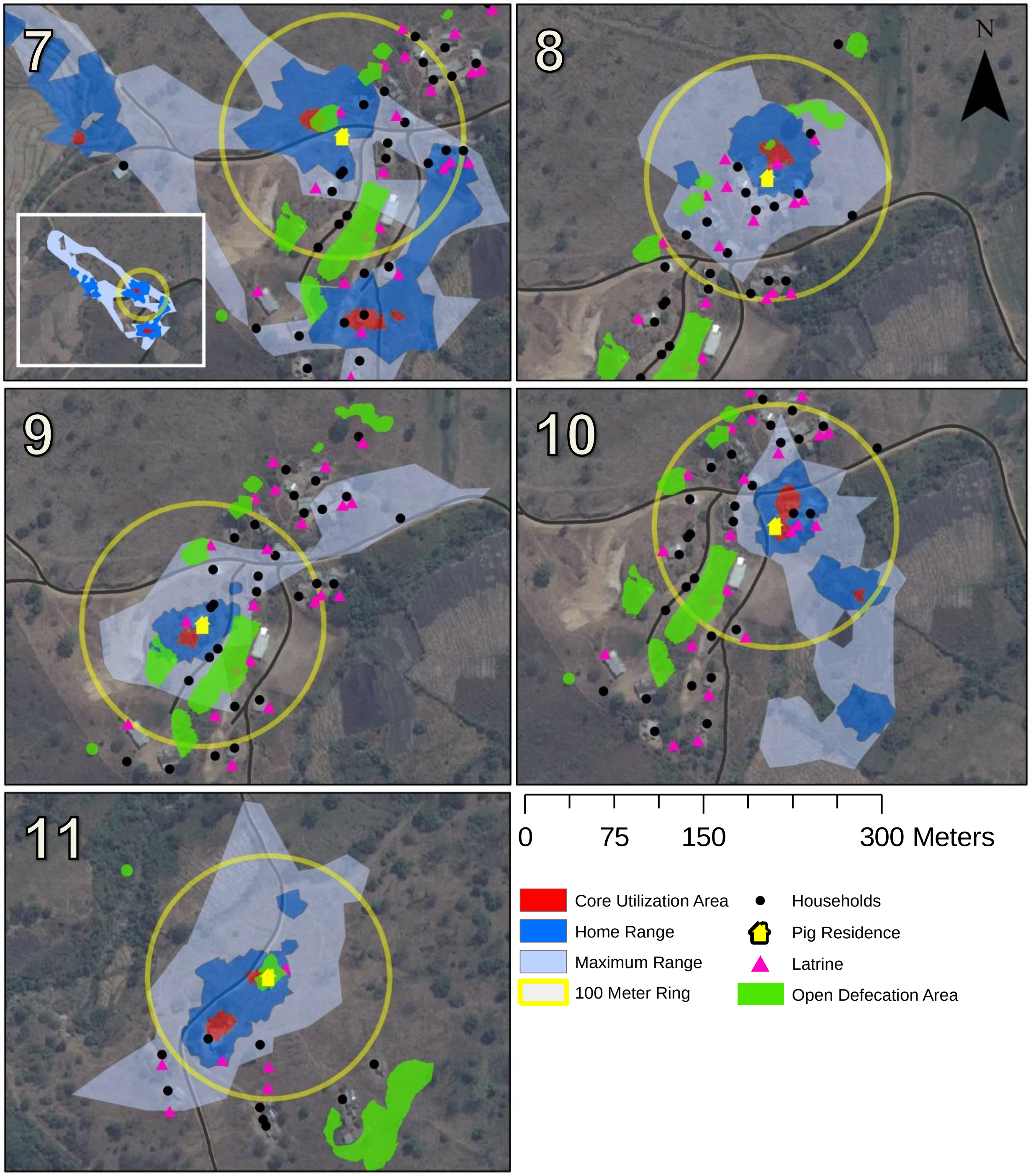

<u>Pigs Tracked (only one pig tracked per household)</u>
Household 7: 12 month-old male
Household 8: 4 month-old male, castrated
Household 9: 4 month-old female
Household 10: 4 month-old male, castrated
Household 11: 4 month-old male, castrated
